# TGLDA: Tail-Guided Low-Density Normal Data Augmentation for Industrial Control Systems

**DOI:** 10.3390/s26185948

**Published:** 2026-09-20

**Authors:** JuHyeon Lee, Ilhwan Ji, Seungho Jeon, Jung Taek Seo

**Affiliations:** 1 Department of Information Security, Gachon University, Seongnam-daero 1342, Seongnam-si 13119, Republic of Korea; 202240226@gachon.ac.kr (J.L.); ilhwan1013@gachon.ac.kr (I.J.); 2 Department of Smart Security, Gachon University, Seongnam-daero 1342, Seongnam-si 13119, Republic of Korea; shjeon90@gachon.ac.kr

**Keywords:** industrial control systems, cybersecurity, data augmentation, rare normal samples, machine learning, sensor measurements

## Abstract

Anomaly detection systems for detecting cyber threats in industrial control systems (ICSs) can suffer performance degradation because rare normal samples may not be sufficiently learned. To address this problem, various data augmentation methods have been studied. However, existing methods may generate data in unknown regions based on model decision boundaries or unnecessarily augment regions that already contain sufficient samples. Many existing methods also require anomalous data. To address these limitations, we propose Tail-Guided Low-Density Normal Data Augmentation (TGLDA), which operates using only normal data. TGLDA consists of a low-density region estimator and a data generator. It identifies low-density regions with relatively few samples in the observed data distribution and selectively augments these regions. TGLDA uses feature-tail seeds as heuristic starting points for generating samples in low-density regions. Experiments on a water treatment cybersecurity dataset show that TGLDA successfully generates data focused on low-density regions compared with other data augmentation methods. When each augmentation method was applied to baseline anomaly detection models, TGLDA achieved the highest Accuracy and F1-score across all models. These results show that TGLDA can mitigate problems caused by a lack of rare normal samples in real-world ICS environments where only normal data are available.

## 1. Introduction

Industrial control systems (ICSs) are used to monitor and control critical processes in national infrastructures, including energy, water treatment, and power systems [1]. Traditionally, ICSs have operated in isolation from external networks. However, increasing integration with information technology (IT) has exposed them to various cyber threats [2]. To address these threats, anomaly detection has been widely studied for identifying abnormal behavior in ICSs [3]. Anomaly detection methods learn normal data patterns and detect data that deviate from these patterns. This approach is suitable for ICS environments where anomalous data are limited. [4]. However, if rare normal samples are not sufficiently represented in the training data, detection models may misclassify them as anomalies and produce false positives [5,6]. Data augmentation has therefore been studied to improve anomaly detection performance in ICSs. Do et al. [7] defined decision boundaries as regions where a detection model trained on normal and anomalous data has difficulty classifying samples. They then generated data near these boundaries and used them for model training. Qin et al. [8] randomly generated data using a generative adversarial network (GAN). They selected samples that the discriminator could not clearly classify and used them to strengthen learning near the decision boundaries. Although these methods improve anomaly detection performance, they generate data from the perspective of a detection model rather than the observed data distribution. As a result, they may generate data outside the observed distribution or in unknown regions. These methods also require anomalous data to define the decision boundaries. In practical ICS environments, however, collecting anomalous data by intentionally creating abnormal conditions is difficult because it can disrupt operational continuity and availability [9,10]. Some studies, such as Tawfik and Badawy [11], have therefore used generative models to augment normal ICS data. However, because these models learn from the training data distribution, they mainly generate samples in high-density regions where clusters are already well formed. Such methods have limited ability to address the lack of rare normal samples described above. Therefore, a data augmentation method is needed that identifies low-density regions containing rare normal samples using only normal data and efficiently generates valid normal samples in these regions without relying on a detection model.

For these reasons, we propose Tail-Guided Low-Density Normal Data Augmentation (TGLDA). TGLDA aims to augment rare samples in low-density regions of normal data. TGLDA consists of a k-nearest neighbors (kNN) [12]-based low-density region estimator and a Soft Actor–Critic (SAC) [13]-based data generator. Existing data augmentation studies assume environments where both normal and anomalous data are available and generate rare samples by identifying decision boundaries. However, these methods are difficult to apply when only normal data are available, which is often the case in ICS environments. In addition, existing methods may generate data outside the observed data distribution. To address these problems, the kNN-based low-density region estimator maps normal data into a latent space and calculates distances between normal samples to identify regions with relatively low density. In this way, the estimator identifies low-density regions containing rare samples based on the observed data distribution when only normal data are available and provides these regions to the data generator. The SAC-based data generator generates data in the low-density regions identified by the estimator. Specifically, SAC starts from a real latent vector and moves the sample toward a target low-density region. It then verifies whether the generated sample belongs to the low-density region. This reduces the expansion of data into unknown regions unrelated to the normal data distribution. To generate data efficiently, TGLDA uses feature-tail seeds as starting points. These seeds are likely to be closer to low-density regions than samples located in densely populated regions. Therefore, this approach is more efficient than random seed selection for SAC. Finally, TGLDA fuses the generated data with real data from the low-density region to prevent large deviations from the characteristics of real normal data. It then verifies whether the fused data belong to the low-density region. Through this process, TGLDA selectively supplements low-density normal regions with insufficient training data while preserving the distributional characteristics of real normal data. Therefore, it can alleviate the insufficient learning of rare normal samples by anomaly detection models.

Experiments on a water treatment cybersecurity dataset showed that TGLDA was more effective at generating data in low-density regions than other data augmentation methods. In addition, when each augmentation method was applied to four anomaly detection models, TGLDA achieved the highest Accuracy and F1-score across all models. The false positive rate (FPR) was also reduced in three of the four models. The main contributions of this paper are as follows:

- We propose TGLDA, which generates rare normal samples in low-density regions for ICS environments where only normal data are available.
- TGLDA consists of a low-density region estimator and a data generator. It uses feature-tail seeds as starting points to generate data more efficiently than random seeds.
- TGLDA adjusts the step size in the latent space to prevent generated data from deviating substantially from the observed data distribution and verifies whether they reach the target low-density region. It then fuses the generated data with real normal data to adjust their values and verifies again whether the final data satisfy the low-density condition.
- Experiments on a water treatment cybersecurity dataset showed that TGLDA was more effective at generating data in low-density regions than other augmentation methods. Anomaly detection models using TGLDA also achieved the highest Accuracy and F1-score.

The remainder of this paper is organized as follows. Section 2 reviews studies on data augmentation for ICSs. Section 3 describes the operation of TGLDA. Section 4 evaluates the effectiveness of TGLDA using the dataset. Finally, Section 5 concludes this paper.

## 2. Related Work

This section reviews studies on data augmentation for ICSs. Although data augmentation can serve various purposes, we focus on methods applicable to anomaly detection. Existing studies are categorized into Synthetic Minority Oversampling Technique (SMOTE)-based data augmentation, generative model-based data augmentation, and model loss-based data augmentation.

### 2.1. SMOTE-Based Data Augmentation

SMOTE-based data augmentation has mainly been used to address class imbalance. Zhang et al. [14] applied SMOTE and Borderline-SMOTE to address data imbalance in ICSs and increase the diversity of minority-class samples. The augmented data were then used to train an intrusion detection system (IDS), reducing the misclassification of minority-class samples. Gu et al. [15] proposed a Data Expansion Intrusion Detection System (DEIDS) by applying a reconstructed convolutional neural network and a data expansion algorithm called CenterBorderline SMOTE to an IDS. DEIDS addressed the shortage of attack data by expanding the available dataset. Jiang and Chen [16] extracted key features from ICS data using a denoising autoencoder (DAE) and applied SMOTE for oversampling. They then used XGBoost to classify the data. Li et al. [17] proposed a contrastive learning-based IDS and augmented the data using SMOTE, random noise, and sequence inversion. These studies can augment minority-class samples in ICSs, but they require environments with at least two classes. Therefore, they are difficult to apply in ICS environments where only the normal class is available.

### 2.2. Generative Model-Based Data Augmentation

Generative model-based data augmentation has mainly been used to generate data that reflect feature-specific patterns. Zhou et al. [18] and Yuan et al. [19] used GANs to generate attack data and expand the available attack datasets. Sha et al. [20] proposed a GAN-based adaptive attack sample expansion algorithm for ICSs. They classified the feature variation patterns of attack samples into three types: oscillation, step, and pulse patterns. They then generated attack samples corresponding to each pattern. This allowed them to generate data similar to real attack samples. Cai et al. [21] proposed a one-dimensional convolutional Wasserstein generative adversarial network (1D CWGAN)-based intrusion detection method for ICSs. The 1D CWGAN combines a one-dimensional convolutional neural network (1D CNN) and a Wasserstein GAN (WGAN) to generate network attack samples. The method identified minority attack sample types that caused performance degradation and augmented these samples in a balanced manner to improve detection Accuracy. Han and Gim [22] generated a large amount of synthetic time-series data using a semi-supervised generative adversarial network (SGAN) to address data scarcity in ICS anomaly detection. Experiments using the augmented data for additional training showed improvements in F1-score and Accuracy across various models. Feng et al. [23] proposed Chip-Based Federated Learning (CGFL) to address data distribution imbalance and client class imbalance. CGFL generated a balanced dataset for each client to improve the performance of the global model. Ferdousi et al. [24] generated additional data using a variational autoencoder (VAE) to train a railway defect classifier. The additional data reduced the reconstruction loss of the model. Jeon and Seo [25] proposed an attention-based variational recurrent autoencoder for data generation. Their method learned the long- and short-term temporal dependencies in ICS data and generated synthetic ICS time-series data. These studies can improve IDS performance through data augmentation. However, methods that augment attack data are difficult to apply in ICS environments where only normal data are available. In addition, existing methods that augment normal data generally generate data across the overall normal data distribution. Therefore, they have difficulty directly addressing performance problems caused by rare samples.

### 2.3. Model Loss-Based Data Augmentation

Model loss-based data augmentation generates samples that are difficult for a model to distinguish as normal or anomalous and augments data near the model decision boundary. Castellini et al. [26] proposed an adversarial example generation method for a hidden Markov model (HMM)-based anomaly detector. Their method uses the anomaly score of the detector to move generated data toward the decision boundary at which the data are classified as anomalous. Samples located before the boundary are then added as normal samples. This process improved anomaly detection performance. Anthi et al. [27] and Mustafa et al. [28] used Jacobian-based Saliency Map attacks to generate adversarial samples and analyze classification behavior. They applied small perturbations to input features based on the decision boundaries of supervised learning models to generate samples that cause misclassification. These samples were then used for additional training, improving the robustness and performance of the supervised models. These studies improve model performance by generating data from the perspective of the detection model. However, this approach may generate data in unknown regions where no real data exist and depends on models trained with both normal and anomalous data. Therefore, it is difficult to apply these methods in ICS environments where only normal data are available.

### 2.4. Limitations of Existing Studies

Although existing studies augment data using various approaches, they have several limitations when applied to practical ICS environments. First, SMOTE-based methods alleviate data imbalance by interpolating between existing minority-class samples. Therefore, they cannot be applied when only the normal class is available [14,15,16,17]. Similarly, methods designed to generate attack samples learn the distributions and feature variation patterns of collected attack data and then expand these distributions. Thus, they require attack data for training [18,19,20,21]. However, obtaining anomalous data by conducting attacks in real ICS environments is nearly impossible. Real-world ICS environments must ensure high availability and operational continuity. Therefore, intentionally conducting attacks to collect data may cause abnormal operation or interruptions in actual processes, making it difficult to collect sufficient attack data in operational environments. In addition, real-world ICSs are large-scale cyber-physical systems, and simulators or testbeds cannot fully reproduce the scale, physical characteristics, and operating conditions of actual systems. Therefore, obtaining data that sufficiently represent the statistical characteristics of real-world attacks is also challenging. For these reasons, it is necessary to consider real-world ICS environments in which only normal data are available. Therefore, SMOTE-based methods and attack sample generation methods are difficult to apply in practice. Model loss-based methods can generate samples that are difficult for a model to distinguish by using misclassification information or the decision boundaries of a trained detection model [26,27,28]. However, these methods depend on a pre-trained detection model, and the locations and characteristics of the generated samples are determined by the loss and decision boundaries of a specific model. In other words, they generate samples from the perspective of a specific model rather than based on the distribution of real normal data. Therefore, the generated samples may not sufficiently reflect the characteristics of normal data that can occur in real environments. Methods that augment normal data using Gaussian noise or generative models mainly focus on applying random modifications to existing data or generating samples similar to the overall normal data distribution [17,22,23,24,25]. Therefore, these methods have difficulty identifying relatively rare or low-density regions within the normal data distribution that may be misclassified by detection models. They also have difficulty selectively augmenting only these regions. To address these limitations, a data augmentation method is needed that uses only normal data to identify low-density regions in the normal data distribution and selectively generates valid rare normal samples in these regions.

## 3. Proposed Methodology

This section describes the proposed TGLDA method. Section 3.1 describes the overall structure of TGLDA. Section 3.2 describes the kNN-based low-density region estimator, and Section 3.3 describes the SAC-based data generator.

### 3.1. Overview

**Figure 1:**
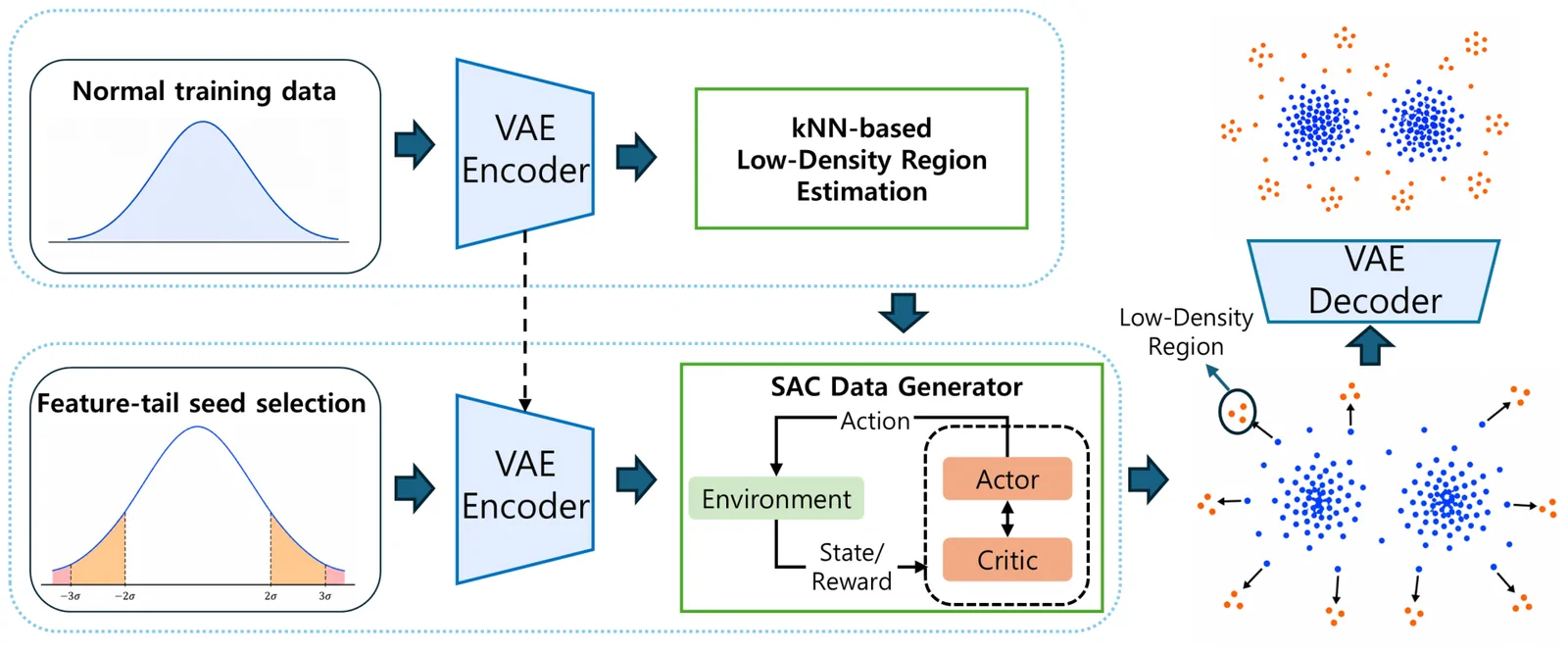
Overview of TGLDA.

Figure 1 shows an overview of TGLDA. TGLDA consists of a kNN-based low-density region estimator and an SAC-based data generator.

The kNN-based low-density region estimator and SAC-based data generator operate in a lower-dimensional latent space obtained through a VAE encoder. We select a VAE to jointly represent the multivariate temporal information within each window and decode new latent vectors into time-series samples. Unlike a standard AE, a VAE learns to reconstruct each input from latent vectors sampled from a regularized distribution rather than only from a single deterministic encoding. This encourages consistent reconstruction around the encoded normal samples, helping the decoder handle new latent vectors produced by SAC-based movement. The kNN-based low-density region estimator calculates kNN distances for all normal data samples. Existing normal data augmentation methods mainly generate data randomly or in regions that are difficult for a model to classify. These methods may unnecessarily generate data in regions where samples are already densely clustered or generate data in unknown regions where no actual data exist. In contrast, the kNN-based low-density region estimator uses kNN distances to identify regions with low density in the actual training data. This approach can identify potential regions where rare samples may be misclassified as anomalies because the model has not learned them sufficiently. This allows TGLDA to determine where additional data should be generated. The SAC-based data generator uses feature-tail samples beyond ±2σ as starting points and moves them toward the low-density regions identified by the estimator. Feature-tail samples beyond ±2σ may be closer to low-density regions than samples within large clusters. Therefore, using these tail samples as starting seeds can generate data more efficiently than random seed selection. SAC stochastically adjusts the movement direction through residual actions, while the step size is controlled by $\eta_{t}$. TGLDA also applies a generation success condition, fusion with real data, and a low-density region check to prevent the generated data from deviating substantially from the observed data distribution. Finally, the generated data are reconstructed to the original dimension through the VAE decoder and used to train the anomaly detection model.

**Definition 1**  (Problem Definition)**.** *Given a normal training dataset $X=\{x_{1},\ldots ,x_{n}\}$ and an augmentation ratio r∈(0,1), TGLDA $G_{\mathrm{TGLDA}}$ is defined as a method that generates a new normal dataset $\tilde{X}$ satisfying the following conditions:*

(1) $$\tilde{X}=G_{\mathrm{TGLDA}}\left(X,r\right),\;\left|\tilde{X}\right|=\left\lfloor rn\right\rfloor$$

(2) $$s_{K}\;\left(f_{\mathrm{VAE}}^{\mathrm{enc}}\left(\tilde{x}\right);Z\right)\geq Q_{q},\;\forall \;\tilde{x}\in \tilde{X}$$

*Here, $f_{\mathrm{VAE}}^{\mathrm{enc}}$ denotes the VAE encoder that maps normal data into the latent space, and $Z={\left\{f_{\mathrm{VAE}}^{\mathrm{enc}}\left(x_{i}\right)\right\}}_{i=1}^{n}$ denotes the set of latent vectors of the normal training data. $s_{K}\left(\cdot ;Z\right)$ denotes the kNN-based low-density score computed with respect to Z, and $Q_{q}$ denotes the empirical q-quantile of these scores. Therefore, TGLDA aims to generate data located in low-density regions of the normal data in the latent space and construct the augmented dataset $X^{\mathrm{aug}}=X\cup \tilde{X}$ by combining the generated data with the original normal data.*

**Training Process of TGLDA.** Given $X=\{x_{1},\ldots ,x_{n}\}$, the VAE $f_{\mathrm{VAE}}$ is trained on $x_{i}$ to generate the reconstructed data ${\hat{x}}_{i}$.

(3) $$z_{i}=f_{\mathrm{VAE}}^{\mathrm{enc}}\left(x_{i}\right),\;{\hat{x}}_{i}=f_{\mathrm{VAE}}^{\mathrm{dec}}\left(z_{i}\right)$$

Here, $f_{\mathrm{VAE}}^{\mathrm{enc}}$ and $f_{\mathrm{VAE}}^{\mathrm{dec}}$ denote the VAE encoder and decoder, respectively. The VAE is trained to jointly minimize the reconstruction error between the input and reconstructed data and the KL divergence [29] for regularizing the latent distribution.

(4) $$L_{\mathrm{VAE}}=\frac{1}{n}\sum_{i=1}^{n}\left[{∥x_{i}-{\hat{x}}_{i}∥}_{2}^{2}+D_{\mathrm{KL}}\left(q_{\phi }\left(z_{i}|x_{i}\right)\|N\left(0,I\right)\right)\right]$$

After training $f_{\mathrm{VAE}}$, the parameters of $f_{\mathrm{VAE}}^{\mathrm{enc}}$ and $f_{\mathrm{VAE}}^{\mathrm{dec}}$ are fixed. The normal training data *X* are then passed through $f_{\mathrm{VAE}}^{\mathrm{enc}}$ to construct the latent vector set *Z* as follows.

(5) $$Z=\left\{z_{1},\ldots ,z_{n}\right\},\;z_{i}=f_{\mathrm{VAE}}^{\mathrm{enc}}\left(x_{i}\right)$$

The low-density region estimator does not require a separate parameter training process. Instead, it uses the normal training latent set *Z* as the reference set for neighbor search and estimates the density of surrounding normal latent vectors. Based on this density, it identifies the general normal region $I_{q}$ and the low-density normal region $B_{q}$. During training, the SAC-based data generator $G_{\mathrm{SAC}}$ learns a policy $\pi_{\theta }\left(a_{t}|o_{t}\right)$ that moves from a starting point $z_{0}\in I_{q}$ toward a real normal latent vector $b^{*}\in B_{q}$ used as the target reference point. The policy optimizes the following objective to jointly maximize the cumulative reward obtained during movement and action diversity:

(6) $$\pi_{\theta }^{*}=\arg\max_{\pi_{\theta }}E_{\pi_{\theta }}\left[\sum_{t=0}^{T-1}\gamma^{t}\left(r_{t}+\alpha H\left(\pi_{\theta }\left(\cdot |o_{t}\right)\right)\right)\right]$$

Here, $o_{t}$, $a_{t}$, $r_{t}$, γ, and α denote the current state provided to the policy, the residual action used to adjust the movement direction, the reward for the movement, the discount factor, and the coefficient controlling action diversity, respectively. After training $G_{\mathrm{SAC}}$, the policy parameters are fixed. Next, $G_{\mathrm{SAC}}$ selects the set of normal data that belong to $I_{q}$ and contain features beyond ±2σ as the candidate rare normal starting-point set $S_{2\sigma }$. The set $S_{2\sigma }$ is transformed by the fixed encoder into the following latent vector set.

(7) $$Z_{2\sigma }=\left\{f_{\mathrm{VAE}}^{\mathrm{enc}}\left(x\right)|x\in S_{2\sigma }\right\}$$

Starting from $Z_{2\sigma }$, the trained policy generates the latent vector set $\tilde{Z}$ that reaches the low-density normal region. The generated latent vectors $\tilde{Z}$ are then passed through the fixed decoder to reconstruct the candidate generated data ${\tilde{X}}_{\mathrm{raw}}$.

(8) $${\tilde{X}}_{\mathrm{raw}}=f_{\mathrm{VAE}}^{\mathrm{dec}}\left(\tilde{Z}\right)$$

### 3.2. kNN-Based Low-Density Region Estimator

This section describes the kNN-based low-density region estimator, which identifies regions with relatively low density in the latent space. The estimator maps the multivariate features of normal time-series data into the latent space using the VAE encoder. It then uses the average kNN distance to determine whether a sample is located in a relatively sparse region. Specifically, the estimator uses the average distance from each normal latent vector to its *K* nearest normal latent vectors, excluding itself, as the low-density score. It then separates the general normal region from the low-density normal region based on the empirical *q*-quantile of the scores computed for all normal latent vectors. Algorithm 1 describes the operation of the proposed kNN-based low-density region estimator.

First, $f_{\mathrm{VAE}}^{\mathrm{enc}}$ transforms the *i*th training sample $x_{i}$ into an *m*-dimensional latent vector. This produces the latent set *Z* consisting of normal latent vectors $z_{i}\in R^{m}$. The low-density score of each latent vector $z_{i}$ is calculated based on its kNN distances to surrounding normal latent vectors. If $z_{i}$ itself is included in the kNN search, a sample with zero distance may be selected as a neighbor. This can underestimate the distances to surrounding samples. Therefore, TGLDA searches for the *K* nearest normal latent vectors $N_{K}^{\left(i\right)}$ from $Z_{-i}$, which excludes $z_{i}$. The average Euclidean distance [30] between $z_{i}$ and the latent vectors $z_{j}$ in $N_{K}^{\left(i\right)}$ is then used as the low-density score $s_{K}\left(z_{i}\right)$. The low-density scores of all normal latent vectors form the set $S_{K}$. A smaller $s_{K}\left(z_{i}\right)$ indicates that normal latent vectors are located closer to $z_{i}$. In contrast, a larger $s_{K}\left(z_{i}\right)$ indicates that $z_{i}$ is farther from its surrounding normal latent vectors and is therefore located in a relatively sparse region of the distribution of normal latent vectors. Thus, TGLDA does not simply treat samples located at the edge of the latent space as boundary samples. Instead, it estimates low-density regions based on the local distribution density of the observed normal data. TGLDA determines $Q_{q}$, corresponding to the *q*-quantile of the distribution of the low-density score set $S_{K}$. By adjusting *q*, the low-density criterion can be determined relative to the data distribution. Based on $Q_{q}$, samples with low-density scores below $Q_{q}$ are assigned to the general normal region $I_{q}$, whereas those with scores greater than or equal to $Q_{q}$ are assigned to the low-density normal region $B_{q}$. Therefore, $B_{q}$ does not represent an unknown space where normal data do not exist. Instead, it represents a region that contains actual normal training data but has a relatively low density of surrounding normal samples. The SAC-based data generator then uses $B_{q}$ as the target region for data generation. Lemma 1 shows that the kNN-based local density of samples in $B_{q}$ cannot exceed a certain upper bound, indicating that the region is relatively sparse in surrounding normal samples.
**Algorithm 1:** kNN-Based Low-Density Region Estimation
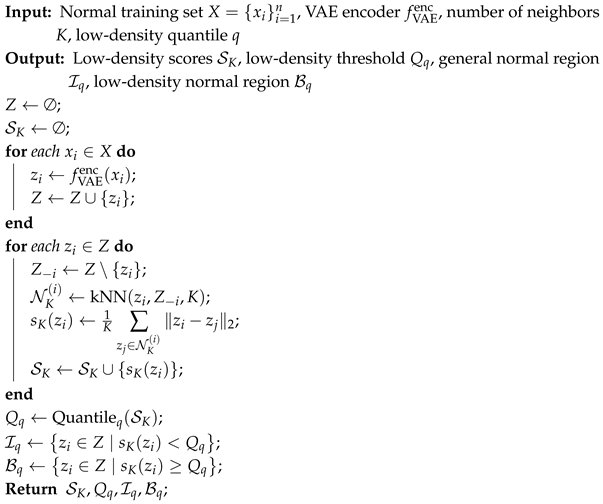

**Lemma 1** 
(Local Density Upper Bound under the Average kNN Distance Criterion)**.** *Given an arbitrary latent vector z and a normal latent vector set Z, suppose that the average kNN distance score satisfies the following condition:*

(9) $$Q_{q}\leq s_{K}\left(z;Z\right)$$

*Let $V_{m}$ denote the volume of the m-dimensional unit ball. The kNN-based local density estimate of z is defined as follows:*

(10) $${\hat{p}}_{K}\left(z;Z\right)=\frac{K}{\left|Z\right|V_{m}{\left(\max_{z_{j}\in \mathrm{kNN}\left(z,Z,K\right)}{∥z-z_{j}∥}_{2}\right)}^{m}}$$

*Then, ${\hat{p}}_{K}\left(z;Z\right)$ satisfies the following upper bound:*

(11) $${\hat{p}}_{K}\left(z;Z\right)\leq \frac{K}{\left|Z\right|V_{m}Q_{q}^{m}}$$

*Therefore, a latent vector with an average kNN distance greater than or equal to $Q_{q}$ has a kNN-based local density bounded above by Equation (11). This indicates that the proposed average-distance criterion selects regions in the normal latent space where surrounding normal samples are relatively sparse.*

**Proof.**  According to the average-distance calculation in Algorithm 1, $s_{K}\left(z;Z\right)$ is the average distance from *z* to its *K* nearest normal latent vectors. Therefore, this average cannot exceed the largest neighbor distance.

(12) $$s_{K}\left(z;Z\right)\leq \max_{z_{j}\in \mathrm{kNN}\left(z,Z,K\right)}{∥z-z_{j}∥}_{2}$$

From Equation (9), the following relation holds:

(13) $$Q_{q}\leq s_{K}\left(z;Z\right)\leq \max_{z_{j}\in \mathrm{kNN}\left(z,Z,K\right)}{∥z-z_{j}∥}_{2}$$

Because the latent dimension *m* is positive and $Q_{q}>0$, raising both sides to the power of *m* and taking reciprocals gives the following relation:

(14) $$\begin{array}{c} Q_{q}^{m}\leq {\left(\max_{z_{j}\in \mathrm{kNN}\left(z,Z,K\right)}{∥z-z_{j}∥}_{2}\right)}^{m}\\ \frac{1}{{\left(\max_{z_{j}\in \mathrm{kNN}\left(z,Z,K\right)}{∥z-z_{j}∥}_{2}\right)}^{m}}\leq \frac{1}{Q_{q}^{m}} \end{array}$$

Applying this relation to Equation (10) gives

(15) $$\begin{array}{cc} {\hat{p}}_{K}\left(z;Z\right)\; & =\frac{K}{\left|Z\right|V_{m}{\left(\max_{z_{j}\in \mathrm{kNN}\left(z,Z,K\right)}{∥z-z_{j}∥}_{2}\right)}^{m}}\\ \; & \leq \frac{K}{\left|Z\right|V_{m}Q_{q}^{m}} \end{array}$$

   □

For $z_{i}\in B_{q}$ in Algorithm 1, setting $z=z_{i}$ and $Z=Z_{-i}$ in Lemma 1 gives $|Z_{-i}|=n-1$. Therefore, the following holds:

(16) $${\hat{p}}_{K}\left(z_{i};Z_{-i}\right)\leq \frac{K}{\left(n-1\right)V_{m}Q_{q}^{m}},\;\forall z_{i}\in B_{q}$$

Therefore, $B_{q}$ is not simply a set of samples with large average kNN distances. It represents a region in which the local density of surrounding normal samples is bounded above by a certain level.

### 3.3. SAC-Based Data Generator

This section describes the SAC-based data generator, which generates new data in low-density normal regions using tail samples as starting points. SAC learns a policy that moves real normal latent vectors toward low-density normal regions. Algorithm 2 describes the process of generating low-density normal data.
**Algorithm 2:** Tail-Guided Low-Density Normal Data Generation Using SAC
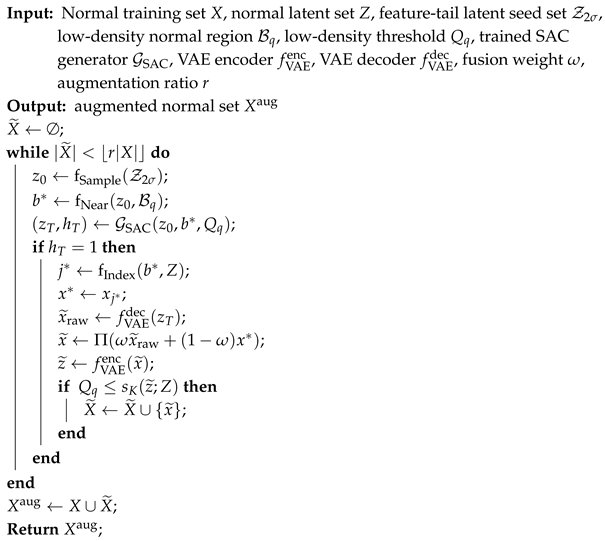


#### 3.3.1. SAC Policy Training for Low-Density Region Movement

$G_{\mathrm{SAC}}$ is an SAC generator trained to move latent vectors from the general normal region $I_{q}$ toward the low-density normal region $B_{q}$. During SAC training, a latent vector $z_{0}$ is selected from $I_{q}$ as the starting point. The nearest low-density normal reference point $b^{*}\in B_{q}$ is then used as the movement target. The reference point $b^{*}$ is a real normal latent vector in the normal latent set *Z*. From the current latent vector $z_{t}$, the distance $D_{t}$ to $b^{*}$ and the movement direction $u_{t}$ are calculated using the Euclidean distance [31].

(17) $$D_{t}={∥b^{*}-z_{t}∥}_{2},\;u_{t}=\left\{\begin{array}{cc} \frac{b^{*}-z_{t}}{D_{t}}, & D_{t}>0\\ 0, & D_{t}=0 \end{array}\right.$$

Here, $\rho_{t}$ and $\rho^{*}$ denote the percentile ranks of the kNN scores of $z_{t}$ and $b^{*}$, respectively, within the overall normal score distribution. The SAC state $o_{t}$ consists of the current latent vector, target direction, distance to the target, current low-density level, and target low-density level. $D_{\mathrm{scale}}$ denotes the normalization factor used to scale the distance to the target reference point.

(18) $$o_{t}=\left[z_{t},u_{t},\frac{D_{t}}{D_{\mathrm{scale}}},\rho_{t},\rho^{*}\right]$$

The SAC actor receives $o_{t}$ and stochastically selects an action $a_{t}$. The action $a_{t}$ is used as a residual action that adjusts the target direction $u_{t}$. The actual movement direction $v_{t}$ and the next latent vector $z_{t+1}$ are calculated as follows.

(19) $$v_{t}=\mathrm{Normalize}\left(u_{t}+\lambda_{a}a_{t}\right),\;z_{t+1}=z_{t}+\eta_{t}v_{t}$$

Here, $\lambda_{a}$ controls the influence of the action on the target direction, and $\eta_{t}$ controls the step size. If $z_{t}$ continues to move with the same step size after its low-density score $s_{K}\left(z_{t};Z\right)$ reaches $Q_{q}$, it may pass $b^{*}$ and move into an excessively sparse region. Therefore, after entering $B_{q}$, TGLDA reduces $\eta_{t}$ to decrease the step size and explore the region around the target reference point more precisely. The SAC reward $r_{t}$ considers the reduction in distance to the target reference point, the reduction in the difference from the target low-density level, the movement direction, and the action magnitude.

(20) $$r_{t}=w_{D}\left(D_{t}-D_{t+1}\right)+w_{e}\left(e_{t}-e_{t+1}\right)+w_{v}\cos\left(v_{t},u_{t}\right)+R_{s}1_{\mathrm{reach}}-w_{a}{∥a_{t}∥}_{2}-R_{f}1_{\mathrm{timeout}}$$

Equation (20) consists of three movement evaluation terms, one target-reaching reward, and two penalty terms. The first term evaluates the reduction in spatial distance between $z_{t}$ and $b^{*}$ during one step. The second term evaluates the reduction in the difference between their low-density levels. Here, $e_{t}=\left|\rho_{t}-\rho^{*}\right|$ represents the difference between the percentile ranks of the kNN scores of $z_{t}$ and $b^{*}$. The third term evaluates how closely the actual movement direction $v_{t}$ aligns with the target direction $u_{t}$. The fourth term provides a reward when the generated latent vector satisfies the target-reaching condition. The fifth term penalizes excessive actions that substantially change the target direction. The final term is a timeout penalty applied when the target-reaching condition is not satisfied within the maximum number of steps *T*. Based on these terms, SAC learns the policy using the objective in Equation (6).

#### 3.3.2. Feature-Tail-Based Low-Density Normal Data Generation and Validation

The SAC-based data generator uses the trained $G_{\mathrm{SAC}}$ to generate ⌊r|X|⌋ samples, corresponding to an augmentation ratio *r* of the input data. Data generation starts from the feature-tail latent seed set $Z_{2\sigma }$. The ±2σ condition is used to select relatively rarely observed normal samples as starting points. Based on the assumption that feature-tail samples are more likely to be closer to low-density regions than samples located near the center of the normal training data distribution, TGLDA selects a starting point $z_{0}$ from $Z_{2\sigma }$. Here, $z_{0}$ belongs to $I_{q}$ and does not belong to $B_{q}$. The function $f_{\mathrm{Near}}$ then selects the low-density reference point $b^{*}$ closest to $z_{0}$. $G_{\mathrm{SAC}}$ takes $\left[z_{0},b^{*},Q_{q}\right]$ as input and outputs the final latent vector $z_{T}$ and the generation success indicator $h_{T}$. The indicator $h_{T}$ equals 1 only when $z_{T}$ satisfies the low-density criterion, is sufficiently close to the target reference point, and reaches a low-density level similar to that of the target.

(21) $$h_{T}=1\left[Q_{q}\leq s_{K}\left(z_{T};Z\right)\right]1\left[{∥b^{*}-z_{T}∥}_{2}\leq \epsilon_{D}\right]1\left[|\rho_{T}-\rho^{*}|\leq \epsilon_{\rho }\right]$$

Here, $\epsilon_{D}$ and $\epsilon_{\rho }$ denote the tolerances for the distance to the target reference point and the difference in low-density level, respectively. Therefore, a latent vector with $h_{T}=1$ is not simply a point located in an arbitrary empty region with a high kNN score. Instead, it is close to the real normal reference point $b^{*}$ and has a low-density level similar to that of the reference point. If $h_{T}=1$, $f_{\mathrm{Index}}$ finds the index $j^{*}$ of the selected $b^{*}$ in *Z*. Because *X* and *Z* are constructed in the same order, $x_{j^{*}}$ is the original normal data corresponding to $b^{*}$. The latent vector $z_{T}$ is then reconstructed by $f_{\mathrm{VAE}}^{\mathrm{dec}}$ as the candidate generated data ${\tilde{x}}_{\mathrm{raw}}$. The candidate generated data ${\tilde{x}}_{\mathrm{raw}}$ and the real normal reference data $x^{*}$ are combined according to the weight ω. The weight ω controls the balance between preserving the generated features and incorporating the real normal features. Π is a post-processing function that ensures that the fused data satisfy valid normal feature values. Through this process, the discrete features of ${\tilde{x}}_{\mathrm{raw}}$ are mapped to the nearest values among the states actually observed in the training data. TGLDA then re-encodes $\tilde{x}$ and verifies whether $\tilde{z}$ satisfies the low-density condition of having a score greater than or equal to $Q_{q}$. If the condition is satisfied, $\tilde{x}$ is added to $\tilde{X}$. Finally, the original dataset *X* and the generated dataset $\tilde{X}$ are combined to construct the augmented dataset $X^{\mathrm{aug}}$. The augmented dataset $X^{\mathrm{aug}}$ is used to train the anomaly detection model.

TGLDA uses four mechanisms to reduce the deviation of generated data from the normal training data distribution. First, in Equation (19), $\eta_{t}$ is reduced after entering the region defined by $Q_{q}$, allowing finer exploration around $b^{*}$. This prevents $z_{T}$ from making a large movement and passing beyond $b^{*}$. Second, in Equation (21), SAC evaluates the generation success by jointly considering the low-density score, the distance to the target reference point, and the low-density level. Third, the generated data are fused with the real normal reference data $x^{*}$, and Π is applied. This allows ${\tilde{x}}_{\mathrm{raw}}$ to partially reflect the features of $x^{*}$ while correcting invalid values. Finally, $\tilde{x}$ is re-encoded to verify that it still satisfies the low-density condition. Through these steps, TGLDA generates data in low-density normal regions while maintaining the validity of normal features. Low-density normal regions are relatively rarely observed in the training data. Therefore, anomaly detection models may not learn these states sufficiently and may classify normal data as anomalous. In such cases, real normal data may be classified as anomalies or attacks, resulting in false positives. TGLDA generates valid normal data in low-density normal regions so that the model can better learn rare normal states. This can reduce false positives. Theorem 1 shows that the kNN-based local density of the data generated by TGLDA satisfies the upper bound given in Lemma 1.

**Theorem 1** 
(kNN-Based Low-Density Guarantee for Final Generated Data)**.** *Given the final generated dataset $\tilde{X}$, the following holds for any $\tilde{x}\in \tilde{X}$.*

(22) $$\tilde{z}=f_{\mathrm{VAE}}^{\mathrm{enc}}\left(\tilde{x}\right),\;{\hat{p}}_{K}\left(\tilde{z};Z\right)\leq \frac{K}{nV_{m}Q_{q}^{m}},\;\forall \tilde{x}\in \tilde{X}$$

*Therefore, the kNN-based local density of every generated sample finally accepted by TGLDA does not exceed the upper bound and satisfies the low-density condition.*

**Proof.**  According to Algorithm 2, every generated sample that is finally accepted satisfies $Q_{q}\leq s_{K}\left(\tilde{z};Z\right)$. This satisfies the condition $Q_{q}\leq s_{K}\left(z;Z\right)$ in Lemma 1. Therefore, Lemma 1 can be applied by setting $z=\tilde{z}$. In addition, because the normal latent vector set *Z* has size |Z|=n, Equation (22) follows directly from Lemma 1.    □

Therefore, Theorem 1 guarantees that the kNN-based local density of the data finally generated by TGLDA does not exceed the upper bound derived in Lemma 1.

## 4. Experiments

This section evaluates the proposed TGLDA through a series of experiments. Section 4.1 describes the experimental setup, evaluation metrics, and data augmentation baselines. Section 4.2 describes the dataset used in the experiments. Section 4.3, Section 4.4 and Section 4.5 evaluate the effectiveness of TGLDA through a series of experiments and answer the research questions (RQs). We also conduct ablation studies of TGLDA and compare its performance with baseline augmentation methods. The RQs are as follows:

- RQ1: How does TGLDA heuristically generate data in terms of generation cost?
- RQ2: How does TGLDA address the problem of sparse data?
- RQ3: How does TGLDA improve the performance of anomaly detection models?

### 4.1. Experimental Environment

**Experimental Setup.** All experiments were conducted on a 64-bit Ubuntu 24.04.4 LTS system equipped with two Intel(R) Xeon(R) Silver 4216 CPUs, an NVIDIA A100 80GB PCIe GPU, and 64 GB of RAM. TGLDA was implemented in PyTorch, and model training and inference were accelerated using CUDA. The input data were standardized using StandardScaler [32]. The normal training data consisted of a total of 495,000 time points. To capture consecutive time-series characteristics, the window length and stride were set to 10 and 5, respectively. This resulted in a total of 98,999 normal training windows. Data augmentation was performed based on these windows, and the test data used the same input structure. Short windows may not sufficiently capture temporal dependencies. Longer windows contain more temporal information but also increase the amount of information that the VAE must compress and reconstruct. A smaller stride increases overlap between windows, which may introduce unnecessary overhead. In contrast, a larger stride reduces the number of training windows and may limit the representation of rare normal states. Therefore, these settings may affect the preservation of temporal patterns in generated samples and the selection of low-density normal regions for augmentation.

The VAE was designed to transform each time-series window into an m=16-dimensional latent vector and reconstruct a time-series window of the same size as the input through the decoder. The encoder and decoder each consisted of two hidden layers. After VAE training, the parameters of both the encoder and decoder were fixed. The low-density region estimator used the normal latent vectors produced by the fixed VAE encoder. The low-density score of each latent vector was calculated as the average distance to its K=30 nearest neighbors, excluding itself. The low-density threshold was set to the 95th percentile (q=0.95) of the low-density score distribution of the normal training data. The kNN-based low-density region estimator has no trainable parameters. The SAC actor and twin critics were implemented using multilayer perceptron (MLP) networks. The replay buffer size was set to $2\times 10^{5}$, the discount factor to 0.99, the soft-update coefficient of the target network to 0.005, and the maximum number of movement steps to 400. The residual action weight was set to $\lambda_{a}=0.35$. The default latent-space step size was set to $\eta_{t}=1$, and it was reduced to $\eta_{t}=0.25$ after entering the low-density region. The fusion weight between the generated data and the real normal reference data was set to ω=0.75. A final generated sample was accepted only when its kNN score was greater than or equal to $Q_{0.95}$.

The VAE and SAC were trained using the Adam optimizer [33]. The learning rate was set to $10^{-4}$, and the maximum number of epochs was set to 200. Training was terminated if the training loss did not improve for 12 consecutive epochs. The hyperparameters of the SAC reward function $r_{t}$ were set to $w_{D}=4.0$, $w_{e}=20.0$, $w_{v}=0.25$, $w_{a}=0.005$, $R_{s}=5.0$, and $R_{f}=1.0$. The tolerances for the generation success indicator $h_{T}$ were set to $\epsilon_{D}=0.10805$ and $\epsilon_{\rho }=0.005$. Therefore, data generation was considered successful when the generated latent vector had a kNN low-density score greater than or equal to $Q_{0.95}$, a distance to the target reference point of at most 0.10805, and a difference from the target low-density level of at most 0.005. If the target-reaching condition was not satisfied within the maximum number of movement steps T=400, the episode was treated as a timeout.

**Metrics.** The evaluation metrics for TGLDA are divided into two categories: data generation metrics and anomaly detection performance metrics. The data generation metrics evaluate whether generated samples reach the target low-density region and how efficiently they are generated. These metrics are defined as follows:

- Low-Density Arrival Rate (LDAR): LDAR represents the proportion of generated samples whose low-density scores are greater than or equal to $Q_{q}$. A higher LDAR indicates that the generated samples are reliably located in the target low-density region.
- Average Steps: Average Steps represents the average number of movement steps required for successful generation episodes to reach the target low-density region. A lower value indicates that the target region is reached with fewer movements.
- Average Latent Distance: Average Latent Distance represents the average distance traveled by SAC in the latent space from the starting point to the final generated position in successful generation episodes.
- Average Generation Time: Average Generation Time represents the average time required to generate low-density normal data and is used to evaluate the generation efficiency of TGLDA.

To evaluate the performance of the baseline models selected for anomaly detection, we use standard performance metrics [34]. These metrics are defined as follows:

- Accuracy: Accuracy represents the proportion of all samples that are correctly classified as normal or anomalous and reflects the overall classification performance of the model.
- Precision: Precision represents the proportion of actual anomalous samples among all samples detected as anomalous.
- Recall: Recall represents the proportion of actual anomalous samples correctly detected by the anomaly detection model.
- F1-Score: F1-score is the harmonic mean of Precision and Recall and represents anomaly detection performance by considering both metrics.
- ROC-AUC: ROC-AUC measures the ability of a model to distinguish normal and anomalous samples across different detection thresholds.
- FPR: FPR represents the proportion of actual normal samples that are incorrectly detected as anomalous. Because TGLDA supplements training data in low-density normal regions, FPR is used to evaluate its effectiveness in reducing false positives for normal data.

**Data Augmentation Baselines.** To compare the data generation performance of TGLDA, we selected several data augmentation baseline methods. All methods used only the normal training data and generated the same number of samples, which were added to the original normal training data. For the VAE, GAN, MAD-GAN, and ALGAN, which require training, the learning rate was set to $10^{-4}$ and the maximum number of epochs was set to 200. Training was terminated if the training loss did not improve for 12 consecutive epochs.

- Gaussian Noise [35]: New samples were generated by adding independent Gaussian noise $\epsilon ∼N(0,0.05^{2})$ to each continuous feature of the normal training data in the standardized feature space. Gaussian noise was not applied to discrete control features, and their original values were preserved.
- VAE [36]: A new VAE was trained using the normal training data, and latent vectors were sampled from the latent distribution estimated by the encoder. The sampled latent vectors were then passed through the decoder to generate new normal time-series data. The VAE used as a baseline was separate from the VAE used in TGLDA.
- GAN [37]: The generator and discriminator were adversarially trained to learn the latent vector distribution of the normal training data. After training, the GAN generated new latent vectors, which were reconstructed into time-series data in the original feature space.
- BeatGAN [38]: As an interpolation-based time-series augmentation method, we used the temporal transformation technique proposed in BeatGAN. Some time points in the normal training sequences were removed, and the average values of adjacent time points were inserted at other positions to generate temporally modified data while maintaining the original sequence length. The same temporal transformation was applied to all features, and only the data augmentation procedure proposed in BeatGAN was used.
- MAD-GAN [39]: We used an LSTM-based adversarial generation method that learns temporal dependencies in multivariate time-series data. The generator and discriminator were adversarially trained using only normal training data. After training, random noise sequences were provided to the generator to generate new multivariate time-series data. Only the data generation method proposed in MAD-GAN was used.
- ALGAN [40]: We used an Adjusted-LSTM-based adversarial generation method that applies attention to the input and hidden states. The generator and discriminator were trained using normal training data so that attention could reflect relevant information within the time series. After training, new time-series data were generated from random noise sequences. Only the data generation method proposed in ALGAN was used.

### 4.2. Dataset

We used the Secure Water Treatment (SWaT) dataset [41] to evaluate TGLDA from various perspectives. SWaT is a widely used ICS testbed dataset. The SWaT testbed is a scaled-down implementation of a six-stage water treatment process that is autonomously controlled by programmable logic controllers (PLCs) and designed to resemble a real operational environment. Each stage includes sensors that measure water level, flow, pressure, and water quality, as well as actuators such as pumps and valves. The overall process is controlled through PLCs, human–machine interfaces (HMIs), and a supervisory control and data acquisition (SCADA) system. In this study, we used the SWaT dataset collected in 2015. The dataset was collected at one-second intervals for a total of 11 days and consists of seven days of normal operation data and four days of attack scenario data. The normal operation file contains 495,000 time points, while the attack scenario file contains 449,919 time points covering both normal and attack periods. Thus, the two files contain a total of 944,919 time points. We excluded the timestamp and label from the model inputs and used d=51 process features consisting of sensor and actuator measurements. The normal operation dataset, SWaT_Dataset_Normal_v1, was used to train TGLDA and the anomaly detection models. The attack scenario dataset, SWaT_Dataset_Attack_v0, was used for the final evaluation of anomaly detection performance. Because all inputs were constructed using a window length of 10 and a stride of 5, the resulting numbers of training and test windows were 98,999 and 89,981, respectively.

### 4.3. RQ1: Efficiency of Feature-Tail Seeds for Low-Density Data Generation

**Figure 2:**
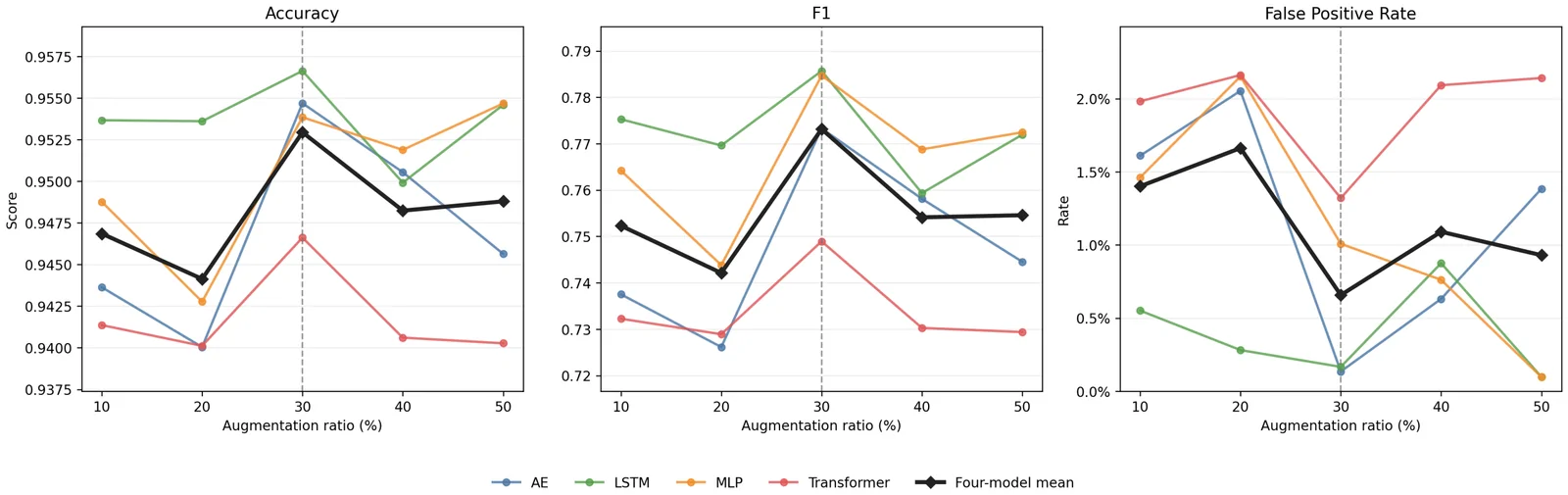
Accuracy, F1-score, and FPR of TGLDA across different data augmentation ratios.

This section addresses RQ1 by experimentally evaluating the cost efficiency of selecting tail seeds to generate data in low-density regions. The generation cost of TGLDA varies with the augmentation ratio. Therefore, we first analyze the performance at different augmentation ratios using baseline anomaly detection models and determine an appropriate ratio. The anomaly detection models are autoencoder (AE) [42], long short-term memory (LSTM) [43], MLP [44], and Transformer [45]. For a fair comparison, all models receive the same time-series windows and perform prediction-error-based anomaly detection by predicting the data at the final time step. The detection threshold for each model is determined using Youden’s J index [46] to balance the true positive rate (TPR) and FPR. Because anomaly detection performance can vary with the augmentation ratio, we first analyze the average performance of TGLDA at augmentation ratios of 10%,20%,30%,40%, and 50%. Figure 2 shows the anomaly detection performance of TGLDA across different data augmentation ratios.

When the data augmentation ratio of TGLDA was 30%, the average Accuracy and F1-score across the four models were the highest, while the average FPR was the lowest. For the individual models, the 30% ratio also showed strong performance in most Accuracy and F1-score results and provided relatively stable performance without being biased toward a specific model or metric. These results indicate that a 30% augmentation ratio is neither too small nor excessive. If the augmentation ratio is too low, low-density normal regions may not be sufficiently supplemented, which can limit the learning effect for rare normal states. In contrast, if the augmentation ratio is too high, the increased proportion of generated data may alter the learning balance of the original normal data distribution or reduce detection sensitivity to anomalous data. In this experiment, the 30% augmentation ratio provided a balance between supplementing low-density normal regions and preserving the original data distribution. In ICS environments where large volumes of data are continuously collected, even small performance improvements may help reduce the costs of unnecessary responses. In this study, we conducted experiments for each model under the same settings. We selected a 30% augmentation ratio based on the balance of Accuracy, F1-score, and FPR observed across the four models, rather than relying solely on small differences in Accuracy. The same augmentation ratio was applied to all data augmentation methods in the subsequent comparison experiments. Because the appropriate augmentation ratio may vary depending on the environment and dataset, it should be empirically determined for each setting.

To efficiently generate data in low-density regions, TGLDA uses samples containing feature-tail values as starting points. Table 1 compares Average Steps, Average Latent Distance, and Average Generation Time for random seeds and tail seeds to evaluate the efficiency of different seed selection strategies.

**Answer to RQ1)** TGLDA selects feature-tail samples as starting points to heuristically reduce data generation cost. We assume that feature-tail samples are relatively closer to low-density regions because they are more sparsely distributed than samples located near the center of the data distribution. Table 1 provides indirect evidence supporting this assumption. Compared with random seeds, tail-seed strategies require fewer Average Steps, shorter Average Latent Distance, and less Average Generation Time. In TGLDA, only the selection of the starting point for data generation changes according to the seed strategy. All seed strategies use the same model trained under the same settings; therefore, changing the seed strategy does not introduce additional training cost. The seed strategy affects the inference cost during the data generation stage of TGLDA, including Average Steps, Average Latent Distance, and Average Generation Time. Average Generation Time represents the average total time required to generate the complete set of low-density rare normal samples for the specified augmentation ratio. Therefore, Table 1 compares the inference cost of the data generation stage under different seed strategies using the same trained TGLDA model. A two-sample t-test showed that the reductions in Average Steps, Average Latent Distance, and Average Generation Time for the ±2σ tail-seed strategy were statistically significant compared with random seed selection (p<0.05). This indicates that the tail seeds are located relatively closer to low-density regions. The ±3σ tail seeds require fewer steps, shorter distances, and less generation time than the ±2σ tail seeds. TGLDA excludes tail seeds that already belong to the low-density region $B_{q}$ and uses only tail seeds in the general normal region $I_{q}$ as starting points. Accordingly, the numbers of available ±3σ and ±2σ tail seeds are 7595 and 36,974, respectively. Although the ±3σ tail seeds are more cost-efficient, the number of available starting points is substantially reduced. This may cause generated data to concentrate in specific low-density regions. Therefore, in this study, TGLDA generates data using ±2σ tail seeds, which provide a more evenly distributed set of starting points.

### 4.4. RQ2: Low-Density Normal Data Augmentation

**Figure 3:**
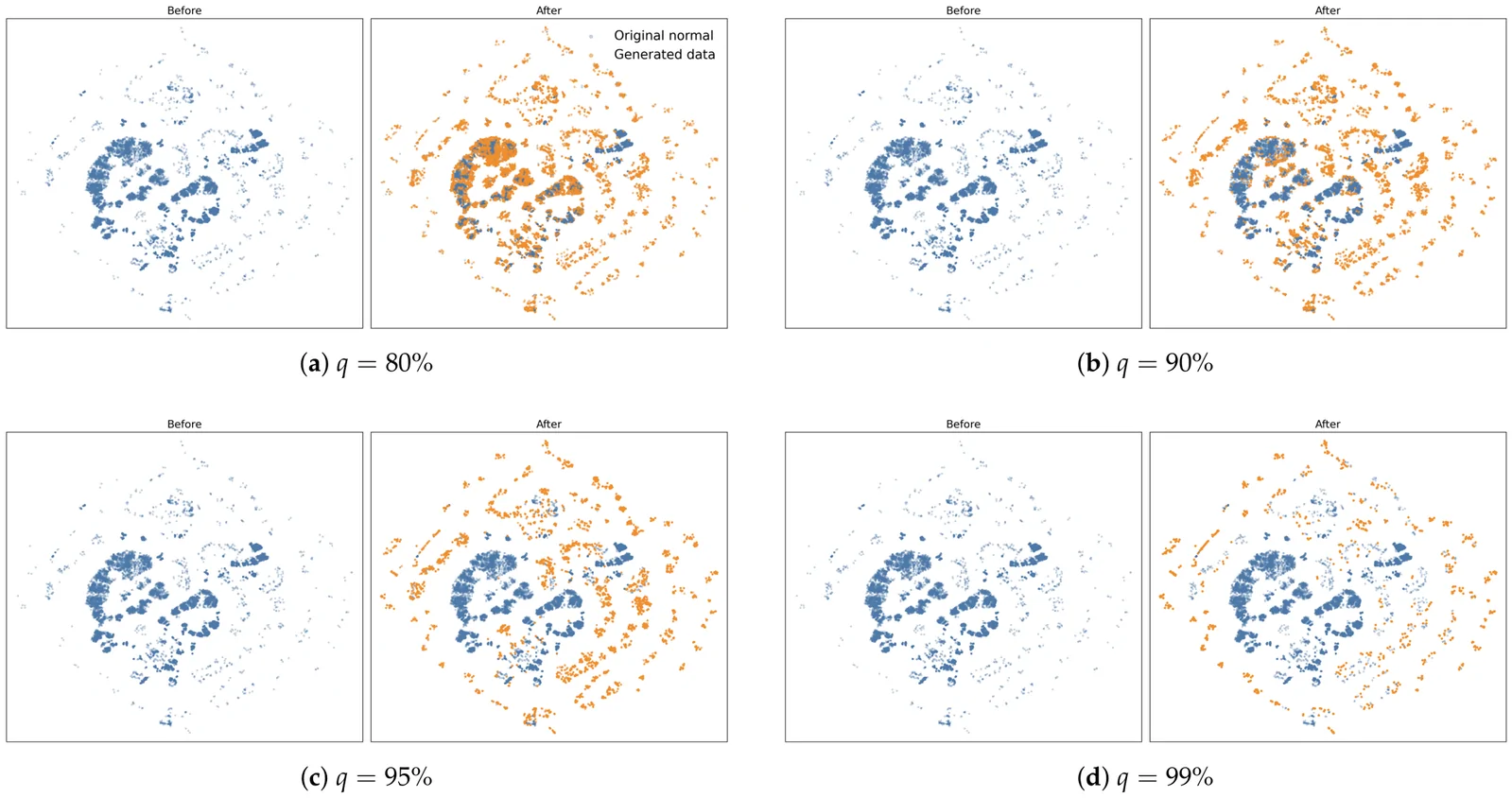
Visual comparison of data generation across different low-density quantiles.

This section addresses RQ2 by experimentally evaluating whether TGLDA alleviates the lack of rare samples by augmenting data in low-density regions. TGLDA defines low-density regions based on the upper tail of the average kNN distance distribution using *q* as the threshold quantile. Therefore, *q* is an important factor because it affects the characteristics of the data generated by TGLDA. Figure 3 visualizes the data generated by TGLDA for different values of *q* using t-SNE [47]. For this comparison, the augmentation ratio was fixed at 30%, and ±2σ tail samples were used as starting points.

TGLDA exhibits different data generation patterns depending on the low-density quantile *q*. At q=80%, many generated samples appear not only in low-density regions but also in high-density clusters where sufficient data are already concentrated. Samples are also generated in empty regions between different clusters, causing previously separated clusters to become connected. This occurs because a relatively wide region is classified as low-density when *q* is low. At q=90%, the range of generated data is reduced compared with q=80%. However, some samples are still generated near the edges of well-formed clusters. This may unnecessarily expand the boundaries of clusters that already contain sufficient samples or alter the shape of the existing normal data distribution. At q=99%, the low-density criterion becomes too strict. As a result, generated samples are concentrated in a few extremely sparse regions, while data generation in other low-density regions is limited. In contrast, at q=95%, TGLDA reduces unnecessary generation in high-density clusters and mainly generates data in low-density regions where normal data are sparsely distributed in the t-SNE space. Therefore, the visual analysis indicates that q=95% provides a relatively appropriate criterion by suppressing unnecessary augmentation in high-density regions while supplementing diverse low-density regions. Because this experiment qualitatively compares the distributions of generated data across different values of *q*, we further evaluate the suitability of each *q* value by using the generated data to train anomaly detection models and comparing their performance. Table 2 shows the anomaly detection performance when the data generated under each low-density quantile are additionally used for model training.

Overall, q=95% showed the most stable anomaly detection performance. At q=95%, the average Accuracy and F1-score across the four models were 0.9530 and 0.7732, respectively, which were the highest among all low-density quantiles. The average FPR was 0.6573%, which was the lowest, and the average ROC-AUC was also the highest at 0.8251. For the individual models, q=95% achieved the highest Accuracy and F1-score for AE, LSTM, MLP, and Transformer. It also achieved the lowest FPR for AE, LSTM, and Transformer. For some individual metrics, the MLP achieved its lowest FPR at q=99%, while the highest ROC-AUC values for MLP and Transformer were obtained at q=80% and q=90%, respectively. However, these results were limited to specific metrics of individual models. In contrast, q=95% showed consistently strong performance across most metrics and models. This is because a low threshold may unnecessarily augment regions that already contain sufficient normal samples, whereas an excessively high threshold may overly restrict the low-density regions available for augmentation. Therefore, considering both the visual analysis and anomaly detection performance, we set q=95% as the criterion for defining low-density regions in this study.

Additionally, we conduct an ablation study to evaluate the effectiveness of the data fusion strategy in TGLDA. TGLDA generates the final data by fusing the generated data with the characteristics of real data. The purpose of this strategy is not to directly improve anomaly detection performance. Instead, it serves as a correction process to mitigate excessive deviation of the generated data from the real normal distribution. Through the data fusion strategy of TGLDA, the generated data can become more similar to the real distribution. Table 3 compares the data distributions before and after applying data fusion with the real distribution. The evaluation metrics include the average kNN distance, the distance between kNN score distributions, the overlap between Kernel Density Estimation (KDE) [48] distributions in the Principal Component Analysis (PCA) [49] space, and the Low-Density Arrival Rate of each data sample. For all metrics, values closer to those of the real low-density data indicate greater similarity to the real distribution.

The data fusion strategy of TGLDA adjusts the generated data to become more similar to the real low-density data distribution. As shown in Table 3, the data generated with data fusion showed a distribution closer to the real low-density data than those generated without data fusion. The average kNN distance was 3.3152 without data fusion and increased to 3.3236 after fusion, becoming closer to 3.3359 of the real low-density normal data. In addition, the Wasserstein distance between the kNN score distributions decreased from 0.03056 to 0.02334, while the KDE distribution overlap in the PCA space increased from 97.14% to 97.23%. The Low-Density Arrival Rate also increased from 98.27% to 100.00%, indicating that all generated samples satisfied the low-density region condition after fusion. These results show that the data fusion strategy is effective in reducing the difference between the generated data and the real low-density normal data distribution while preserving the low-density characteristics of the generated data. Therefore, the data fusion strategy of TGLDA serves as a correction process to mitigate excessive deviation of the generated data from the real normal data distribution.

**Figure 4:**
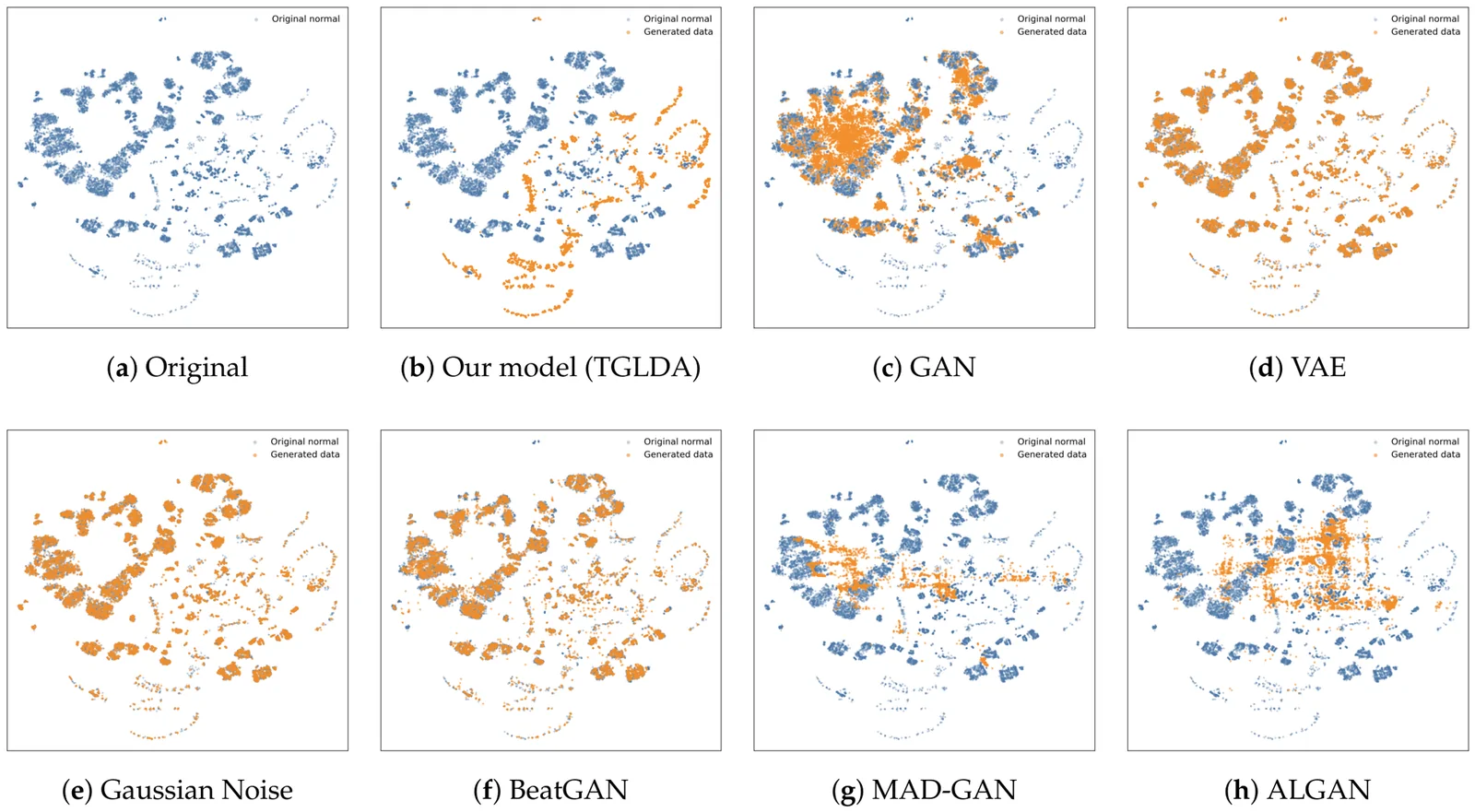
Visual comparison of generated data between TGLDA and baseline augmentation methods.

We visually compare TGLDA with other data augmentation baselines to further evaluate the effectiveness of low-density region augmentation. Figure 4 visualizes the data generated by each method using t-SNE.

**Figure 5:**
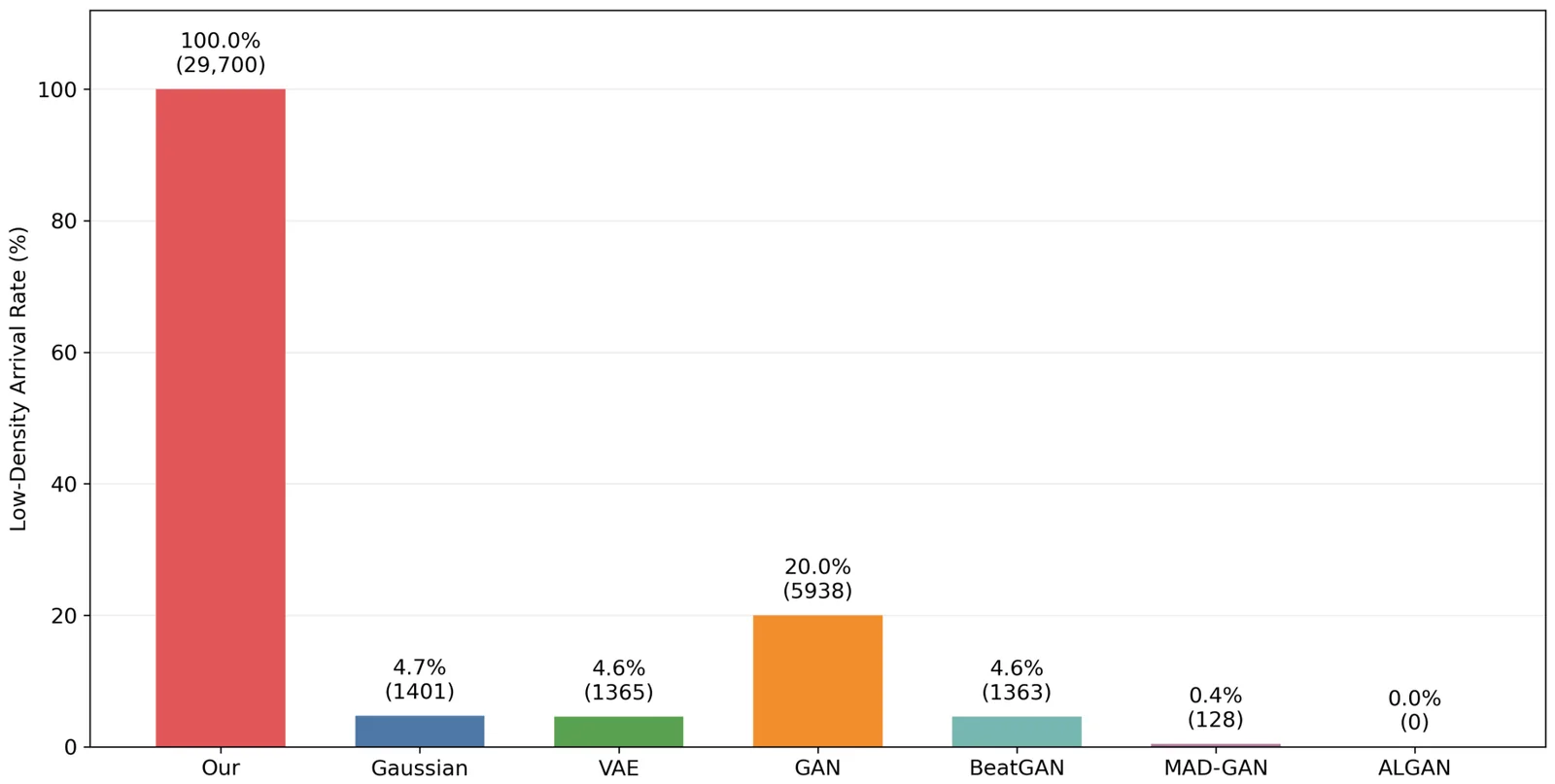
Low-density arrival rate of generated data across different augmentation methods.

**Answer to RQ2)** TGLDA generates data by focusing on regions with low density, where the number of samples is small. Therefore, TGLDA generates data similar to the rare samples located in low-density regions. Figure 4 visually compares the distributions of data generated by TGLDA and the baseline augmentation methods. The data distribution before applying each augmentation method shows that some regions form dense clusters, while others are more widely spread and have relatively low density. In low-density regions, relatively few normal samples are observed, resulting in visible gaps in the data distribution. TGLDA augments rare samples in these low-density regions. In this experiment, TGLDA generated data mainly in actual low-density regions where normal samples exist and reduced unnecessary augmentation in high-density regions. This shows that TGLDA does not simply expand the overall normal data distribution, but selectively augments regions where normal data are relatively insufficient. Gaussian Noise, VAE, and BeatGAN generated samples across the existing normal data distribution and also added samples to already dense clusters. In the visualization, the samples generated by GAN, MAD-GAN, and ALGAN tended to concentrate in specific regions or extend into areas that did not overlap with existing normal samples. In contrast, TGLDA selectively augmented low-density regions containing real normal samples, thereby supplementing regions where normal data were relatively insufficient. Figure 5 shows the proportion of generated data that reached low-density regions for each data augmentation method.

TGLDA achieved an LDAR of 100%, as all 29,700 generated samples satisfied the low-density condition. In contrast, Gaussian Noise, VAE, and GAN achieved LDARs of 4.7%, 4.6%, and 20.0%, respectively. BeatGAN, MAD-GAN, and ALGAN achieved LDARs of 4.6%, 0.4%, and 0.0%, respectively. The low LDARs of Gaussian Noise, VAE and BeatGAN result from generating samples across the overall normal data distribution rather than focusing on low-density regions. These methods generate relatively few samples in low-density regions, where fewer data are available, while generating more samples in high-density regions. In the visualization, GAN, MAD-GAN, and ALGAN tended to concentrate generated samples in specific regions. However, their LDARs differed substantially. GAN achieved the highest LDAR among the baseline methods, whereas MAD-GAN and ALGAN generated few or no samples satisfying the low-density condition. These methods do not explicitly target low-density normal regions, which limits their ability to selectively supplement rare normal samples. Overall, these results show that TGLDA specifically generates data in targeted low-density regions compared with the other data augmentation methods. Together with Theorem 1, the experimental results further demonstrate that TGLDA generates data that satisfy the low-density condition.

### 4.5. RQ3: Effect of TGLDA on Anomaly Detection Performance

This section addresses RQ3 by analyzing the effect of TGLDA on anomaly detection performance. Specifically, we train anomaly detection models with additional data generated by TGLDA and the baseline augmentation methods and compare their effects on model performance. As described above, TGLDA selectively generates data in low-density normal regions to supplement areas with insufficient training data. This can help reduce false positives in which actual normal data are incorrectly classified as anomalous. Table 4 shows the Accuracy, F1-score, and FPR of each anomaly detection model after applying the different data augmentation methods. For each model and evaluation metric, the best and second-best results are highlighted in orange and blue, respectively.

**Answer to RQ3)** TGLDA augments rare normal samples so that anomaly detection models can learn these patterns more effectively. This can alleviate model misclassification. As shown in Table 4, TGLDA achieved the highest Accuracy and F1-score for AE, LSTM, MLP, and Transformer. These results demonstrate that the proposed TGLDA provides consistent performance improvements across different models rather than improving the performance of only a single model. For MLP and Transformer, TGLDA also achieved the lowest FPRs of 1.0076% and 1.3201%, respectively. These results show that adding low-density normal data reduced false positives for normal data while improving overall detection performance. For MLP, MAD-GAN and ALGAN slightly reduced FPR relative to no augmentation, while TGLDA achieved the lowest FPR. For AE and LSTM, TGLDA did not achieve the lowest FPR but showed the second-best results. For LSTM, the FPR of TGLDA was 0.1665%, which was higher than the value of 0.1331% obtained by GAN but lower than the value of 0.1842% obtained without augmentation. The detection models adjust their thresholds using Youden’s J index. This approach balances TPR and FPR rather than minimizing FPR alone. Therefore, although TGLDA had a slightly higher FPR than GAN, its higher Accuracy indicates that it missed fewer attacks than GAN. At the same time, TGLDA achieved the highest Accuracy and F1-score. For AE, TGLDA achieved the highest Accuracy and F1-score of 0.9547 and 0.7733, respectively. However, its FPR slightly increased to 0.1348% compared with 0.1098% without augmentation. For AE, this increase in FPR was observed across all augmentation methods, not only TGLDA. One possible reason is that differences in patterns between generated and real normal data affect the representations learned by the AE during compression and reconstruction. This may change the prediction-error distribution of real normal samples and lead to an increase in FPR. Nevertheless, the FPR of TGLDA for AE was lower than those obtained with all other augmentation methods, indicating relatively stable performance compared with the other augmentation methods. Overall, unlike the baseline augmentation methods that do not explicitly target low-density normal regions, TGLDA selectively supplements low-density normal regions with insufficient training data. As a result, TGLDA improved Accuracy and F1-score across all anomaly detection models. It also reduced FPR for some models. These results show that the proposed low-density normal data augmentation can improve the learning of normal patterns across different anomaly detection models rather than being limited to a specific model.

Considering the previous experimental results, TGLDA can identify low-density regions where normal samples are relatively rare and selectively generate rare normal data in these regions. This is particularly useful in ICS environments where collecting attack data is difficult and only normal data are available. Rather than expanding the distribution into new regions where no normal samples have been observed, TGLDA uses low-density regions containing real normal samples as generation targets. This helps preserve the distributional characteristics of the original normal data. By supplementing these regions, anomaly detection models can learn rare normal operating states that are insufficiently represented in the training data. This can help reduce false positives in which normal data are misclassified as anomalies or attacks. These results show that TGLDA provides a normal data augmentation method applicable to various ICS environments without relying on attack data or the decision boundaries of a specific anomaly detection model.

## 5. Conclusions

In this study, we proposed Tail-Guided Low-Density Normal Data Augmentation (TGLDA) to augment rare samples in practical ICS environments where only normal data are available. Existing data augmentation methods may generate new data near unknown model-dependent boundaries or unnecessarily augment regions where clusters are already well formed. In contrast, TGLDA identifies low-density regions containing rare samples in the observed data distribution and selectively augments normal data in these regions. This allows TGLDA to efficiently supplement rare samples that are insufficiently represented in the normal training data. TGLDA also uses feature-tail samples as starting points during data generation. This allows the generated samples to reach low-density regions with shorter movement distances and less generation time than random seed selection. The generated data are then fused with real low-density normal data and verified using the low-density condition. This process prevents the generated data from substantially deviating from the normal training data distribution. Compared with other data augmentation methods, TGLDA generated data by focusing on low-density regions, whereas the baseline methods did not explicitly target low-density regions. Experiments with anomaly detection models showed that TGLDA achieved the highest Accuracy and F1-score for all models. It also reduced the FPR for three models. These results indicate that TGLDA can mitigate the insufficient representation of rare normal samples in practical ICS environments where only normal data are available.

## Figures and Tables

**Table 1:** Comparison of seed selection strategies for low-density data generation at a 30% augmentation ratio.

Seed Strategy	Steps	Latent Distance	Generation Time (s)
Random Seed	23.269	3.198	4.885
±2σ Tail Seed	23.153	3.120	4.669
±3σ Tail Seed	20.665	2.850	4.159

**Table 2:** Anomaly detection performance across different low-density quantiles.

Quantile	Model	Acc.	F1	FPR	ROC-AUC
q=80%	AE	0.9535	0.7693	0.2841%	0.8203
	LSTM	0.9488	0.7533	0.8948%	0.8136
	MLP	0.9318	0.7174	3.7928%	**0.8492**
	Transformer	0.9442	0.7400	1.5672%	0.8156
	Average	0.9446	0.7450	1.6347%	0.8247
q=90%	AE	0.9529	0.7663	0.3355%	0.8203
	LSTM	0.9506	0.7648	0.9401%	0.8150
	MLP	0.9474	0.7590	1.5839%	0.8379
	Transformer	0.9456	0.7447	1.3985%	**0.8180**
	Average	0.9491	0.7587	1.0645%	0.8228
q=95%	AE	**0.9547**	**0.7733**	**0.1348%**	**0.8250**
	LSTM	**0.9566**	**0.7857**	**0.1665%**	**0.8160**
	MLP	**0.9539**	**0.7847**	1.0076%	0.8424
	Transformer	**0.9466**	**0.7489**	**1.3201%**	0.8169
	Average	**0.9530**	**0.7732**	**0.6573%**	**0.8251**
q=99%	AE	0.9525	0.7639	0.3441%	0.8175
	LSTM	0.9470	0.7512	1.3064%	0.8118
	MLP	0.9535	0.7737	**0.5320%**	0.8156
	Transformer	0.9403	0.7282	2.0942%	0.8178
	Average	0.9483	0.7543	1.0692%	0.8157

**Table 3:** Effect of real-data fusion on the similarity between generated data and real low-density normal data.

Evaluation Metric	Real Low-Density Data	Without Data Fusion	With Data Fusion
Average kNN Distance	3.3359	3.3152	**3.3236**
Wasserstein Distance of kNN Score Distributions	0.00000	0.03056	**0.02334**
KDE Distribution Overlap in PCA Space	100.00%	97.14%	**97.23%**
Low-Density Arrival Rate	100.00%	98.27%	**100.00%**

**Table 4:** Anomaly detection performance across different data augmentation methods. Bold values with orange and cyan backgrounds indicate the best and second-best results, respectively.

Method	AE			LSTM			MLP			Transformer		
	Acc.	F1	FPR	Acc.	F1	FPR	Acc.	F1	FPR	Acc.	F1	FPR
No Augmentation	**0.9545**	**0.7716**	**0.1098%**	0.9539	0.7691	0.1842%	0.9480	0.7609	1.5316%	0.9436	0.7373	1.6204%
Gaussian Noise	0.9517	0.7621	0.4850%	0.9478	0.7527	1.1723%	0.9328	0.7279	4.0662%	0.9455	0.7444	**1.4058%**
VAE	0.9525	0.7650	0.3982%	0.9527	0.7701	0.5738%	0.9339	0.7268	3.7158%	0.9415	0.7303	1.8645%
GAN	0.9407	0.7283	1.9690%	**0.9543**	0.7710	**0.1331%**	0.9481	**0.7729**	2.1509%	0.9449	0.7419	1.4767%
BeatGAN	0.9517	0.7621	0.4885%	0.9537	**0.7776**	0.6651%	0.9261	0.7015	4.4639%	0.9424	0.7349	1.8142%
MAD-GAN	0.9450	0.7428	1.4896%	0.9419	0.7312	1.7838%	0.9466	0.7529	1.5063%	0.9365	0.7150	2.4681%
ALGAN	0.9501	0.7570	0.7122%	0.9440	0.7411	1.6892%	**0.9500**	0.7717	**1.4810%**	**0.9457**	**0.7454**	1.4076%
Our (TGLDA)	**0.9547**	**0.7733**	**0.1348%**	**0.9566**	**0.7857**	**0.1665%**	**0.9539**	**0.7847**	**1.0076%**	**0.9466**	**0.7489**	**1.3201%**

## Data Availability

The data presented in this study are available on request from the corresponding author.

## References

[1] Knapp E.D., Langill J.T. (2014). Industrial Network Security: Securing Critical Infrastructure Networks for Smart Grid, SCADA, and Other Industrial Control Systems.

[2] Bhamare D., Zolanvari M., Erbad A., Jain R., Khan K., Meskin N. (2020). Cybersecurity for industrial control systems: A survey. Comput. Secur..

[3] Seo J.K., Lee J., Kim B., Shim W., Seo J.T. (2026). AI-based anomaly detection in industrial control and cyber–physical systems: A data-type-oriented systematic review. Electronics.

[4] Ahmed C.M., MR G.R., Mathur A.P. Challenges in machine learning based approaches for real-time anomaly detection in industrial control systems. Proceedings of the 6th ACM on Cyber-Physical System Security Workshop.

[5] Kim H., Mok J., Lee H., Shin J., Yoon S. CANDI: Curated test-time adaptation for multivariate time-series anomaly detection under distribution shift. Proceedings of the AAAI Conference on Artificial Intelligence, Singapore EXPO.

[6] Chen Z., Xie X., Yang L., Lai J.H. (2025). Hard-normal example-aware template mutual matching for industrial anomaly detection. Int. J. Comput. Vis..

[7] Do H.K.N., Nguyen T., Hassanaly M., Alharbi R., Seo J., Thai M. Swift hydra: Self-reinforcing generative framework for anomaly detection with multiple mamba models. Proceedings of the International Conference on Learning Representations, Singapore EXPO.

[8] Qin Z., Liu Y., Wang T., Chen L., Song Y. (2026). Effective Sample Generation for Industrial Control Systems: A GAN-Based Approach. IEEE Trans. Ind. Inform..

[9] Choi S., Yun J.H., Kim S.K. A comparison of ICS datasets for security research based on attack paths. Proceedings of the International Conference on Critical Information Infrastructures Security.

[10] Ani U.P.D., Watson J.M., Green B., Craggs B., Nurse J.R. (2021). Design considerations for building credible security testbeds: Perspectives from industrial control system use cases. J. Cyber Secur. Technol..

[11] Tawfik M., Badawy W. Adversarial Generation of Modbus TCP Packets Using WGANs for ICS Security Testing. Proceedings of the 2025 International Telecommunications Conference (ITC-Egypt).

[12] Steinbach M., Tan P.N., Wu X., Kumar V. (2009). kNN: k-Nearest neighbors. The Top Ten Algorithms in Data Mining.

[13] Haarnoja T., Zhou A., Abbeel P., Levine S. Soft actor-critic: Off-policy maximum entropy deep reinforcement learning with a stochastic actor. Proceedings of the International Conference on Machine Learning.

[14] Zhang Y., Zhang L., Zheng X. (2024). Enhanced intrusion detection for ICS using MS1DCNN and transformer to tackle data imbalance. Sensors.

[15] Gu H., Lai Y., Wang Y., Liu J., Sun M., Mao B. (2022). DEIDS: A novel intrusion detection system for industrial control systems. Neural Comput. Appl..

[16] Jiang J.R., Chen Y.T. (2022). Industrial control system anomaly detection and classification based on network traffic. IEEE Access.

[17] Li C., Li F., Zhang L., Yang A., Hu Z., He M. (2023). Intrusion detection for industrial control systems based on improved contrastive learning SimCLR. Appl. Sci..

[18] Zhou W., Kong X.m., Li K.l., Li X.m., Ren L.l., Yan Y., Sha Y., Cao X.y., Liu X.j. (2021). Attack sample generation algorithm based on data association group by GAN in industrial control dataset. Comput. Commun..

[19] Yuan L., Yu S., Yang Z., Duan M., Li K. (2023). A data balancing approach based on generative adversarial network. Future Gener. Comput. Syst..

[20] Sha Y., Chen Z., Liu X., Yan Y., Du C., Liu J., Han R. (2022). Adaptive industrial control system attack sample expansion algorithm based on generative adversarial network. Appl. Sci..

[21] Cai Z., Du H., Wang H., Zhang J., Si Y., Li P. (2023). One-dimensional convolutional Wasserstein generative adversarial network based intrusion detection method for industrial control systems. Electronics.

[22] Han C., Gim G. (2025). Time-series-based anomaly detection in industrial control systems using generative adversarial networks. Processes.

[23] Feng S., Gao L., Shi L. (2025). CGFL: A robust federated learning approach for intrusion detection systems based on data generation. Appl. Sci..

[24] Ferdousi R., Yang C., Hossain M.A., Laamarti F., Hossain M.S., Saddik A.E. (2024). Generative model-driven synthetic training image generation: An approach to cognition in railway defect detection. Cogn. Comput..

[25] Jeon S., Seo J.T. (2024). A synthetic time-series generation using a variational recurrent autoencoder with an attention mechanism in an industrial control system. Sensors.

[26] Castellini A., Masillo F., Azzalini D., Amigoni F., Farinelli A. (2023). Adversarial data augmentation for hmm-based anomaly detection. IEEE Trans. Pattern Anal. Mach. Intell..

[27] Anthi E., Williams L., Rhode M., Burnap P., Wedgbury A. (2021). Adversarial attacks on machine learning cybersecurity defences in industrial control systems. J. Inf. Secur. Appl..

[28] Mustafa A., Khan M.T., Umer M.A., Masood Z., Ahmed C.M. Adversarial sample generation for anomaly detection in industrial control systems. Proceedings of the 1st Workshop on Modeling and Verification for Secure and Performant Cyber-Physical Systems.

[29] Joyce J.M., Lovric M. (2025). Kullback–Leibler divergence. International Encyclopedia of Statistical Science.

[30] Faisal M., Zamzami E.M., Sutarman (2020). Comparative analysis of inter-centroid K-Means performance using euclidean distance, canberra distance and manhattan distance. J. Phys. Conf. Ser..

[31] Liberti L., Lavor C. (2017). Euclidean Distance Geometry: An Introduction.

[32] Raju V.G., Lakshmi K.P., Jain V.M., Kalidindi A., Padma V. Study the influence of normalization/transformation process on the accuracy of supervised classification. Proceedings of the 2020 Third International Conference on Smart Systems and Inventive Technology (ICSSIT).

[33] Hospodarskyy O., Martsenyuk V., Kukharska N., Hospodarskyy A., Sverstiuk S. Understanding the Adam Optimization Algorithm in Machine Learning. Proceedings of the 2nd International Workshop on Computer Information Technologies in Industry 4.0 (CITI 2024).

[34] Nguyen T.H.T., Mouksassi M.-S., Holford N., Al-Huniti N., Freedman I., Hooker A.C., John J., Karlsson M.O., Mould D.R., Pérez Ruixo J.J. (2017). Model evaluation of continuous data pharmacometric models: Metrics and graphics. CPT Pharmacomet. Syst. Pharmacol..

[35] Becerra-Suarez F.L., Alvarez-Vasquez H., Forero M.G. (2025). Improvement of bank fraud detection through synthetic data generation with Gaussian noise. Technologies.

[36] Wan Z., Zhang Y., He H. Variational autoencoder based synthetic data generation for imbalanced learning. Proceedings of the 2017 IEEE Symposium Series on Computational Intelligence (SSCI).

[37] Figueira A., Vaz B. (2022). Survey on synthetic data generation, evaluation methods and GANs. Mathematics.

[38] Liu S., Zhou B., Ding Q., Hooi B., Zhang Z., Shen H., Cheng X. (2023). Time series anomaly detection with adversarial reconstruction networks. IEEE Trans. Knowl. Data Eng..

[39] Li D., Chen D., Jin B., Shi L., Goh J., Ng S.K. MAD-GAN: Multivariate anomaly detection for time series data with generative adversarial networks. Proceedings of the International Conference on Artificial Neural Networks.

[40] Bashar M.A., Nayak R. (2025). ALGAN: Time series anomaly detection with Adjusted-LSTM GAN. Int. J. Data Sci. Anal..

[41] Goh J., Adepu S., Junejo K.N., Mathur A. (2017). A dataset to support research in the design of secure water treatment systems. Proceedings of the International Conference on Critical Information Infrastructures Security, Paris, France,
10–12 October 2016.

[42] Wang C., Wang B., Liu H., Qu H. (2020). Anomaly detection for industrial control system based on autoencoder neural network. Wirel. Commun. Mob. Comput..

[43] Feng C., Li T., Chana D. Multi-level anomaly detection in industrial control systems via package signatures and LSTM networks. Proceedings of the 2017 47th Annual IEEE/IFIP International Conference on Dependable Systems and Networks (DSN).

[44] MR G.R., Somu N., Mathur A.P. (2020). A multilayer perceptron model for anomaly detection in water treatment plants. Int. J. Crit. Infrastruct. Prot..

[45] Shang W., Qiu J., Shi H., Wang S., Ding L., Xiao Y. (2024). An efficient anomaly detection method for industrial control systems: Deep convolutional autoencoding transformer network. Int. J. Intell. Syst..

[46] Ruopp M.D., Perkins N.J., Whitcomb B.W., Schisterman E.F. (2008). Youden Index and optimal cut-point estimated from observations affected by a lower limit of detection. Biom. J..

[47] Van der Maaten L., Hinton G. (2008). Visualizing data using t-SNE. J. Mach. Learn. Res..

[48] Chen Y.C. (2017). A tutorial on kernel density estimation and recent advances. Biostat. Epidemiol..

[49] Abdi H., Williams L.J. (2010). Principal component analysis. Wiley Interdiscip. Rev. Comput. Stat..

